# Innovative treatment of hypercapnia with soda lime in COVID-19: a case report

**DOI:** 10.11604/pamj.2023.44.132.32845

**Published:** 2023-03-15

**Authors:** Ali Amoushahi, Pietro Salvatori

**Affiliations:** 1 Department of Anesthesiology and Intensive Care Unit, Isabn-e-Maryam Hospital, Isfahan, Iran,; 2 Head and Neck Surgeon, Ear Nose Throat Department, Humanitas San Pio X Hospital, Milan, Italy

**Keywords:** COVID-19, hypercapnia, soda lime, case report

## Abstract

One of the rare consequences of COVID-19 is increasing blood carbon dioxide, which can lead to unconsciousness, dysrhythmia, and cardiac arrest. Therefore, in COVID-19 hypercarbia, non-invasive ventilation (with Bi-level Positive Airway Pressure, BiPAP) is recommended for treatment. If CO_2_ does not decrease or continues rising, the patient's trachea must be intubated for supportive hyperventilation with a ventilator (Invasive ventilation). The high morbidity and mortality rate of mechanical ventilation is an important problem of invasive ventilation. We launched an innovative treatment of hypercapnia without invasive ventilation to reduce morbidity and mortality. This new approach could open the window for researchers and therapists to reduce COVID death. To investigate the cause of hypercapnia, we measured the carbon dioxide of the airways (mask and tubes of the ventilator) with a capnograph. Increased carbon dioxide inside the mask and tubes of the device was found in a severely hypercapnic COVID patient in the Intensive Care Unit (ICU). She had a 120kg weight and diabetes disease. Her PaCO_2_ was 138mmHg. In this condition, she had to be under invasive ventilation and accept its complication or lethal risk but we decreased her PaCO_2_ with the placement of a soda lime canister in the expiratory pathway to absorb CO_2_ from the mask and ventilation tube. Her PaCO_2_ dropped from 138 to 80, and the patient woke up from drowsiness completely without invasive ventilation, the next day. This innovative method continued until PaCO_2_ reached 55 and she was discharged home 14 days later after curing her COVID. Soda lime is used for carbon dioxide absorption in anesthesia machines and we can research its application in hypercarbia state in ICU to postpone invasive ventilation for treatment of hypercapnia.

## Introduction

Uncontrolled hypercapnia is a life-threatening consequence among severe cases of COVID-19 who are involved in acute respiratory distress syndrome (ARDS). Patients who rapidly deteriorate and develop ARDS require life support with mechanical ventilation. This condition leads to a high rate of mortality in the intensive care unit [1]. Unfortunately, mechanical ventilation can cause pneumothorax and further injury to lung tissue already damaged by COVID-19. The extracorporeal carbon dioxide removal (ECCO_2_R) technique is another way of treating critical patients but this method is very invasive, too [2].

We present the case of a patient with severe ARDS of COVID-19 who was rescued with the utilization of soda lime throughout the expiratory path. A humidifier is incompletely filled with soda lime instead of water and placed in the expiratory way of the ventilation device. It decreased end-tidal CO_2_ and PaCO_2_ in the patient and she was discharged after 14 days from ICU to home without any tracheal intubation or invasive mechanical ventilation. This is a very easy, safe, and low-cost treatment.

## Patient and observation

**Patient information:** the patient is a 38-year-old woman, with a 120kg weight and diabetes disease. She was admitted to the ICU because of ARDS due to COVID-19. Her husband complained about her snoring from sleep sickness. Non-invasive mask ventilation with CPAP and BiPAP portable ventilators failed to decrease her PaCO_2_.

**Clinical findings:** her physical examination showed pulmonary fine crackles. In clinical evaluation, severe hypoxia (Spo2=50-80%), severe hypercapnia (PaCO_2_= 100-138mmHg), and near 90% lung involvement in computed tomography (CT-scan) was seen.

**Timeline of the current episode:** the patient was admitted to COVID-ICU on October 9^th^ 2021, due to hypoxia and hypercapnic (PaCO_2_= 65mmHg) condition. We initiated non-Invasive ventilation with CPAP and then BiPAP portable ventilator. Her CO_2_ was rising and resisted non-invasive ventilation. We changed her ventilation to non-invasive ventilation with a hospital mechanical ventilator in ICU, on October 10^th^ 2021. Because of arise in her PaCO_2_ to 138, we started our innovation. Soda lime is added to the respiratory circle via a canister. After that, her CO_2_ decreased, rapidly (Table 1).

**Table 1:** timeline of current episode

Date	Relevant past medical history and interventions		
8 Nov 2021			
**Date**	**Summaries from initial and follow-up visits**	**Diagnostic Testing**	**Interventions**
9 Nov 2021	The patient admitted to COVID-ICU due to hypoxic and hypercapnic condition	PaCO_2_=67mmHg	20 lit/min oxygen therapy with mask and reserve bag
		SpO_2_=80%	
10 Nov 2021	We administer Non-invasive ventilation with CPAP and BiPAP ventilator.	PaCO_2_=93mmHg	Non-invasive ventilation
		SpO_2_=65%	
11 Nov 2021	Because of raising PaCO_2_ to 138, we initiate our innovative treatment hypercapnia with conduct her expiratory way to soda lime canister	PaCO_2_=138mmHg	Soda lime placing
		SpO_2_=45-50%	
12 Nov 2021	Decreasing CO_2_	PaCO_2_=82 mmHg	Continue soda lime adding
		SpO_2_=50-60%	
14 Nov 2021	Decreasing CO_2_	PaCO_2_=67 mmHg	renew soda limes
		SpO_2_=75-85%%	

**Figure 1:**
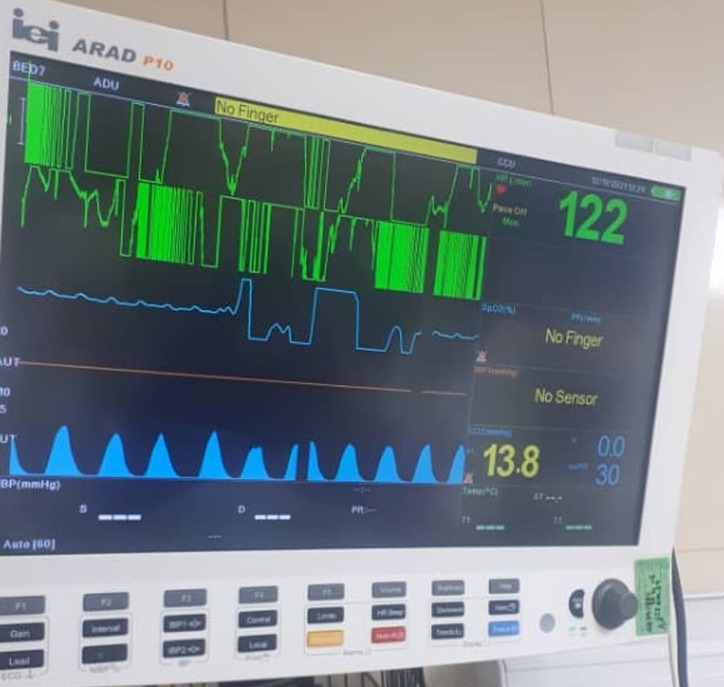
increase of carbon dioxide to 32.7 under the BiPAP mask

**Figure 2:**
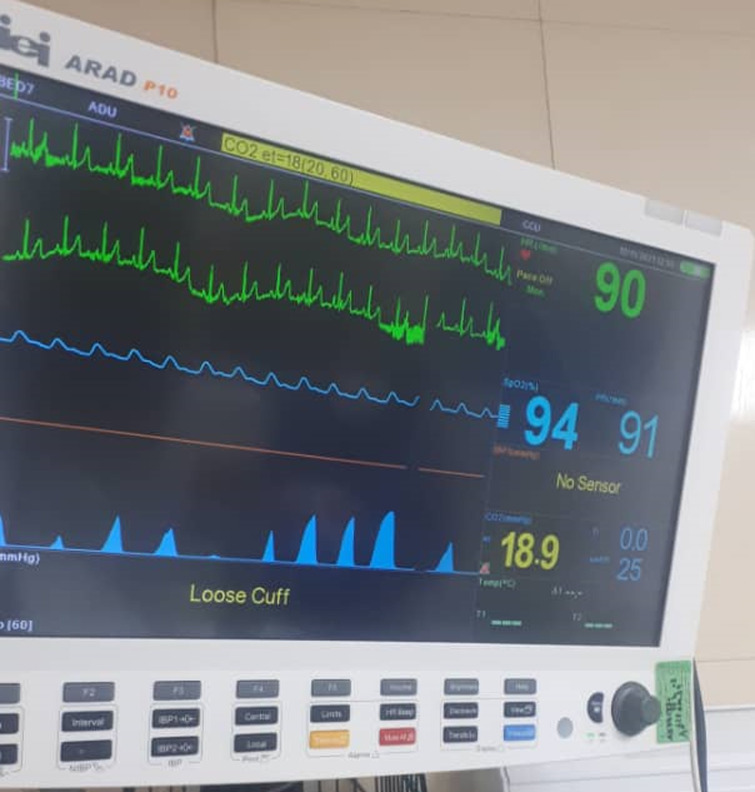
increase of carbon dioxide to 18.9 in the BiPAP tube

**Diagnosis assessment:** her SARS-CoV-2 PCR test was positive and her lung X-rays showed disseminated lung infiltration marked as COVID-19. We measured the carbon dioxide of the airways (mask and tubes of the ventilator) with a capnograph. Capnography showed increased end-tidal CO_2_ inside the mask near 32.7mmHg (Figure 1) and near 19.9mmHg in the tubes (Figure 2) of the BiPAP portable ventilator. The PaCO_2_ was rising in serial blood gases in parallel to the end-tidal CO_2_ of the capnograph.

**Diagnosis:** severe COVID-19, respiratory failure, and hypercapnia. This stage is a poor prognosis.

**Therapeutic intervention:** first intervention was non-invasive ventilation with CPAP and the next step was with the BiPAP portable ventilator. Her PaCO_2_ was rising and refractive non-invasive ventilators. In this condition, she had to be under invasive ventilation and accept its complication and lethal risk but we decreased her PaCO_2_ with the placement of soda lime granules in the expiratory pathway to absorb CO_2_ from the mask and ventilator tube. Soda lime is a carbon dioxide adsorbent used through expiratory tubes of anesthesia machines as an absorber of CO_2_. It is a mixture of sodium, calcium hydroxide, and potassium hydroxide. They combine with exhaled carbon dioxide and produce water, heat, sodium hydroxide, and calcium carbonate.

**Figure 3:**
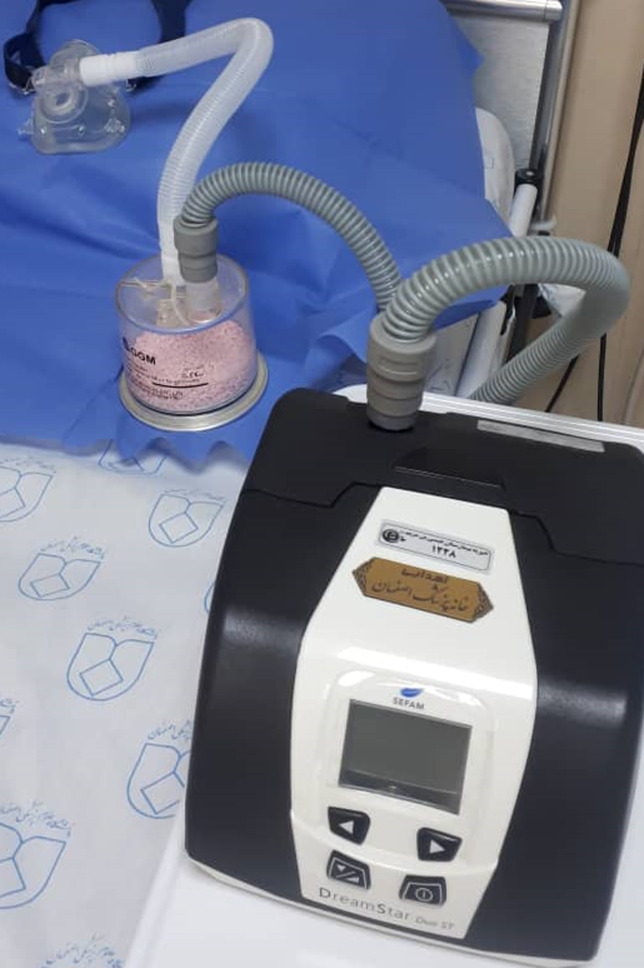
placing a canister of soda lime in the expiration pathway after the mask and before the BiPAP

At first, a humidifier chamber was partially filled with soda lime instead of distilled water. Then, by a short (50cm) duct we connected the exit of the face mask to the soda lime chamber. After that, the respiratory tube was connected from the chamber to the portable BiPAP ventilator (Figure 3). By starting the ventilator, the capnograph showed rapidly decreasing CO_2_ under the mask and within the expiratory tube. PaCO_2_ decreased from 138 to 80 after 8 hours, too, and the patient woke up from drowsiness completely without tracheal intubation. This technique continued every day to achieve PaCO_2_ near 50mmHg. We controlled the color of soda lime to renew it after deactivation, which changes its color from pink to white.

**Follow-up and outcomes:** soda lime decreased the end-tidal CO_2_ and PaCO_2_ of the patient without the need for tracheal intubation and invasive mechanical ventilation or an extracorporeal CO_2_ removal system. The patient was discharged from ICU after 10 days and 4 days later went home while her PaCO_2_ was 51mmHg. One month later she analyzed her blood gasses and found PaCO_2_ 57mmHg at home. She didn't report any respiratory irritation or adverse events. Her inflammatory factors such as C-reactive protein didn't increase.

**Patient perspective:** during this method, she and her husband, and her family were very delighted because of the non-invasive intubation.

**Informed consent:** her husband gave consent to apply a new idea for treatment. She and her husband were informed of the case report, innovation, and the authors' interest in publishing the case.

**Patient consent:** her husband and her family had given their consent for publishing clinical information although there is no patient- identifiable data.

## Discussion

The non-invasive ventilation mask shape shows very small holes and a small exit valve. It is suspicious because of air re-breathing in these devices that we can establish it by capnography. In a tachypneic state, the patient does not have enough time to expel carbon dioxide through the holes of the mask. So it is constantly pushed back into the mask and lungs, and finally, the hypercapnia occurs.

To eliminate this phenomenon, we added carbon dioxide adsorbents like an anesthesia machine [3] instead of invasive hyperventilation or extracorporeal CO_2_ removal. This report describes the successful use of soda lime placing in the expiratory way of non-invasive ventilation on a COVID-19 ARDS patient with multiple risk factors for poor outcomes, including obesity and diabetes. This method has been used in anesthesia machines for many years and has not had any adverse effects. If we absorb the carbon dioxide of the airway while in a re-breathing state, it removes plasma CO_2_ and improves the pH of arterial blood gas.

**Figure 4:**
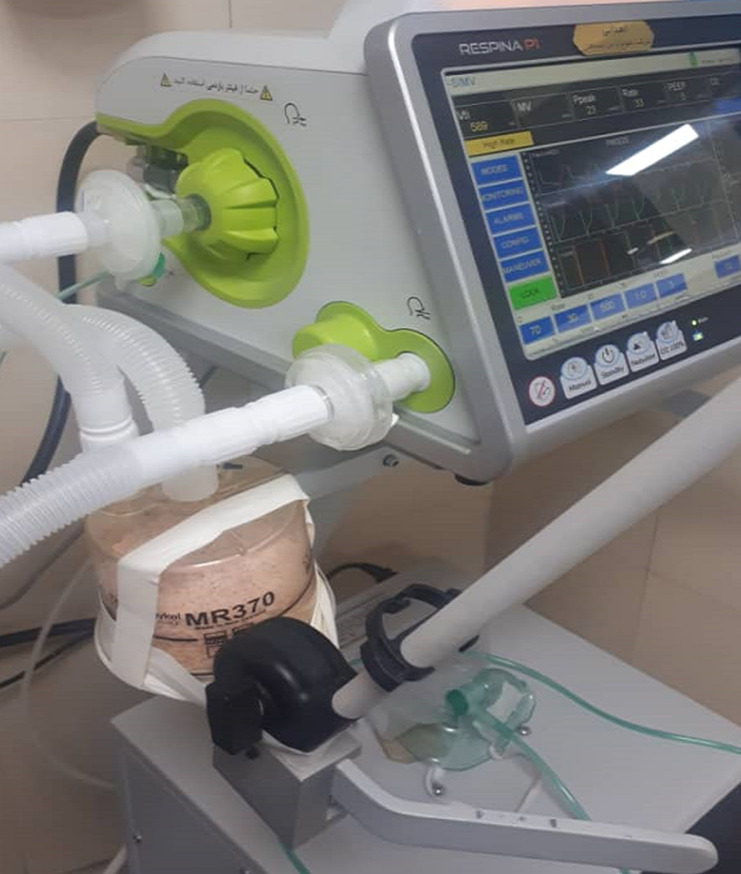
incomplete filling of the humidifier of ventilator with soda lime and placing it in the expiratory pathway

The correct way for placing soda lime in an expiratory way, NOT in an inspirational way, is one limitation because this is a new technique (Figure 4). Pneumothorax, which can occur as a complication of mechanical ventilation or develop spontaneously from COVID-19 infection, has been thought to be a grave prognostic factor [4]. This case shows that putting away tracheal intubation and invasive mechanical ventilation helped the patient survive.

## Conclusion

**Figure 5:**
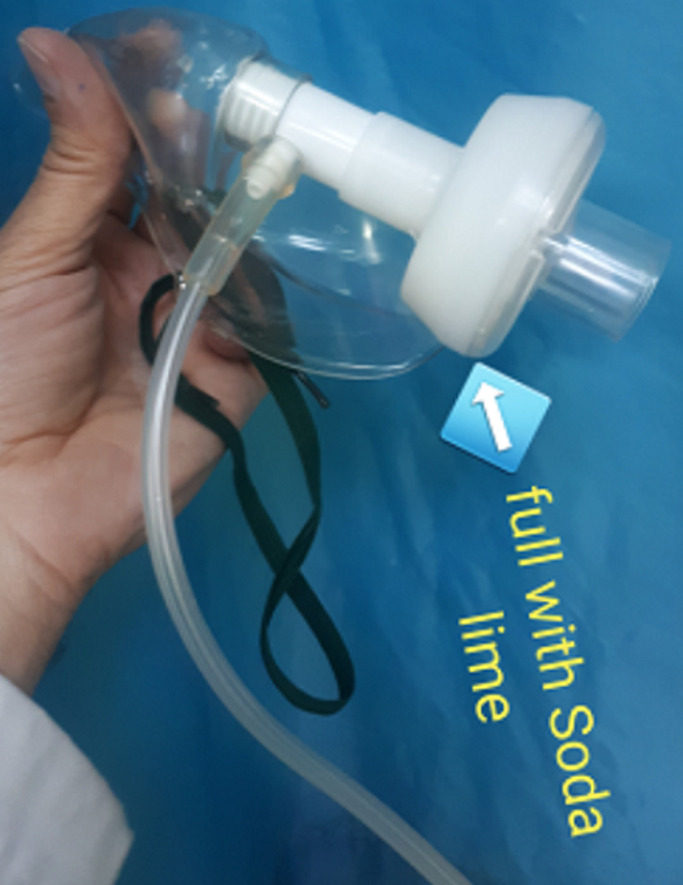
propose making a face mask of oxygen with antibacterial filter and soda lime bottle or filter in the back

Soda lime facilitates effective treatment of a patient with severe refractory hypercapnic status secondary to COVID-19. Conducting an expiratory circle through a soda lime chamber allowed absorption of CO_2_ in non-invasive ventilation and minimized the risk of intubation, need for sedation, pneumothorax, and another lung injury. Since it is easy to prove the re-breathing of CO_2_ in non-invasive ventilation and because invasive ventilation with tracheal intubation is a high-risk treatment in COVID-19; it is recommended to take tracheal intubation one step next to soda lime and advice soda lime at the first step in the list of indications for mechanical ventilation in hypercapnia. In the future, it may be possible to equip oxygen masks with soda lime filters to prevent the re-breathing of CO_2_ (Figure 5).
